# Oxygen persufflation as adjunct in liver preservation (OPAL): Study protocol for a randomized controlled trial

**DOI:** 10.1186/1745-6215-12-234

**Published:** 2011-10-28

**Authors:** Thomas Minor, Carolin Pütter, Anja Gallinat, Claudia Ose, Gernot Kaiser, Andre Scherag, Jürgen Treckmann, Andreas Paul

**Affiliations:** 1 Surgical Research Division, University Clinic of Surgery, Bonn, Germany; 2 Center for Clinical Trials Essen (Zentrum für Klinische Studien Essen - ZKSE) at the Institute for Medical Informatics, Biometry and Epidemiology, University of Duisburg-Essen, Germany; 3 Dpt. for General, Visceral and Transplantation Surgery, University Hospital of Essen, Germany

## Abstract

**Background:**

Early graft dysfunction due to preservation/reperfusion injury represents a dramatic event after liver transplantation. Enhancement of donor organ criteria, in order to cope with the ever increasing donor shortage, further increases graft susceptibility to ischemic alterations.

Major parts of post-preservation injury, however, occur at the time of warm reperfusion but not during ischemic storage; successful reperfusion of ischemic tissue in turn depends on an adequate redox and intracellular signal homeostasis. The latter has been shown experimentally to be favorably influenced by oxygen persufflation within short time spans. Thus viability of marginally preserved liver grafts could still be augmented by transient hypothermic reconditioning ***even after ***normal procurement and static cold storage. The present study is aimed to confirm the conceptual expectations, that hypothermic reconditioning by gaseous oxygen persufflation is a useful method to suppress injurious cellular activation cascades and to improve post-ischemic recovery of marginally preserved liver grafts.

**Methods/Design:**

OPAL is a prospective single center randomized proof of concept study, including two parallel groups in a total of 116 liver transplant patients. The effect of an in hospital treatment of the isolated liver graft by 2 hours of oxygen persufflation immediately prior to transplantation will be assesses as compared to standard procedure (cold storage without further intervention). The primary endpoint is the peak transaminase serum level (AST) during the first three days after transplantation as a surrogate readout for parenchymal liver injury. Other outcomes comprise patient and graft survival, time of intensive care requirement, hepatic tissue perfusion 1h after revascularisation, early onset of graft dysfunction based on coagulation parameters, as well as the use of a refined scoring-system for initial graft function based on a multi-parameter (AST, ALT, Quick and bilirubin) score. Furthermore, the effect of OPAL on molecular pathways of autophagy and inflammatory cell activation will be evaluated. Final analysis will be based on all participants as randomized (intention to treat).

**Trial Registration:**

Current Controlled Trials ISRCTN00167887

## Background

Early graft dysfunction due to preservation/reperfusion injury represents a dramatic event after liver transplantation, affecting long term prognosis of graft viability and patient outcome [1,2].

In face of the growing shortage of donor organs, the acceptance criteria for liver retrieval have been expanded torwards the inclusion of of older donors and the use of 'less than optimal' organs. In doing so, the increased proclivity of these organs to ischemia and cold preservation enhances the risk of primary dys- or even non-function (PNF) of the liver after transplantation. While the number of donors aging > 65 years has increase more than ten-fold from 1991 to 2001 in the United Network for Organ sharing as well as the European Liver Transplant registry [3], increased donor age has been incriminated in large series as a prominent factor associated with decreased graft survival [4,5]. A multivariate analysis of 1148 donor livers showed significantly decrease 3 year survival (62%) in livers originating from donors of > 65 years compared to 75% standard criteria donor grafts [6]. Likewise, moderate to severe degrees of fat accumulation, which was present in 12% of all donor livers, was found an independent predictor of increased preservation injury [4]. Thus the challenges rise concerning maintenance and/or restoration of tissue integrity during the storage period.

An elegant means to do so lies in the provision of oxygen during storage using gaseous oxygen application through the vascular system. The method of venous systemic oxygen persufflation [7] is a simple and versatile way to regenerate cellular energy dependent pathways under conditions of relatively low metabolic work load. Any improvement in the preservation of grafts, which were procured from extended criteria donors and/or experienced extended times of preservation represent a valuable advance to enlarge the total number of viable donor organs and to circumvent the need of re-transplantation.

Earlier experimental studies have suggested that major parts of the post preservation injury occur at the time of warm reperfusion but not during ischemic storage and that deleterious priming of the graft during ischemia can be abrogated prior to transplantation by dynamic revitalization techniques at the end of cold storage [8,9], even in liver grafts which were donated after cardiac death [10].

Since successful reperfusion of ischemic tissue most likely depends on an adequate redox and intracellular signal homeostasis, which is favorably influenced by oxygen persufflation within short time spans, viability of long preserved liver grafts is improved by transient hypothermic reconditioning (HR) prior to implantation.

The optimal treatment time for hypothermic reconditioning of initially cold stored livers by gaseous oxygen persufflation has previously been investigated in a preclinical large animal model. One hour of HR already notably improved functional outcome upon reperfusion, but the maximal therapeutic effect was obtained after 2 hours [11]. Thus, 2 hours of end-ischemic oxygen persufflation of cold stored porcine livers significantly improved survival after transplantation [12].

The technique of vascular oxygen persufflation not only maintains cell and organ integrity and function but also enables (to some extend) the repair of damaged structures and restoration of cellular ion and signal homeostasis.

Moreover, it has shown to reverse ischemia induced breakdown of cellular autophagy [13], thus restoring the cell's regenerative capacity upon reperfusion to remove damaged organelles and recycle denaturated proteins [14,15].

Owing to its simplicity and ease of application, oxygen persufflation appears to be a particularly convenient approach, already shown to be safe and easily applicable to the human situation [16].

Based on these promising results, the present clinical trial protocol was designed as a final Proof-of-Concept study aiming to establish oxygen persufflation as valuable adjunct in clinical liver preservation.

## Methods/Design

This is a single center, randomized, controlled, single blind clinical Proof-of Concept study.

Using a two parallel arms approach (treatment/control) the question will be addressed whether a hypothermic reconditioning protocol using 2h of gaseous oxygen persufflation of the isolated liver graft immediately prior to transplantation will improve early graft function upon reperfusion and mitigate adverse effects associated with preservation/reperfusion injury as compared to standard care (simple cold storage without further intervention).

Only patients who meet all inclusion/exclusion criteria are considered to be included in the trial. The inclusion criteria are:

• Men and women beyond 18 years of age

• Resident in Germany

• Scheduled for first liver transplantation and graft already available

• Patient is willing and able to attend regular follow up examinations

• Written informed consent

### Inclusion criteria for the donor liver

• Donor grafts that are offered to the local Transplant clinic for Implantation, i.e. 'organ rescue offers', or donor age above 65

### Main exclusion criteria

Patients presenting with any of the following are not included in the trial:

• Listed as high urgency (HU)

• Participation in this study at an earlier time

• Simultaneous participation in other clinical trial

• Positive test for HIV

• Pregnant or nursing

All patients are observed for seven days following transplantation on a daily basis. Follow up includes additional observations on the day of discharge and 3 month after transplantation. Patients are followed until 3 month after the last patient is randomized for this trial and are asked to attend clinical routine follow up subsequent to termination of the study.

The schedule for study related activities and data collection is listed in table 1.

**Table 1:** Flow chart

	screening	waiting- phase	preoperative	OP	postoperative								follow up (3 month)
						
					D 1		D3	D3	D4	D5	D6	discharge	
Informed consent	X												
In-/ex-clusion criteria	X		X										
Demography	X												
Medical history	X												
Randomisation			X										
BQS/complications			X	X								X	X
Postoperative dialysis					X	X	X	X	X	X	X	X	
Time of mechanical ventilation					X	X	X	X	X	X	X	X	
Laboratory parameters	X		X		X	X	X	X	X	X	X	X	X
SAE			X	X	X	X	X	X	X	X	X	X	
Death				X	X	X	X	X	X	X	X	X	X
Retransplantation					X	X	X	X	X	X	X	X	X

### Objectives and endpoints

It is the aim of the present study to demonstrate the efficacy of short-term oxygen persufflation as end-ischemic adjunct in liver preservation to improve early graft recovery after transplantation.

Serum peaks of liver transaminases are expected to be surrogates of the extent of reperfusion injury and represent the most commonly used parameters for the progression of liver related disease. They correlate well with parenchymal graft injury [17], usually associated with initial liver dysfunction after transplantation in clinical studies [4,18]. Therefore the peak value of systemic aspartate aminotransferase (AST) during the first three days after transplantation has been chosen as the most important primary endpoint of this study. In considering the serum levels during a period of three days it is thought that a more solid basis will be obtained for the judgment of the individual liver under investigation and to alleviate possible skews of single measurements.

Secondary outcomes are death and retransplantation (3 month mortality) as well as time of ICU-stay, time of ventilation and haemodialysis, complication rate, hepatic tissue perfusion 1h after revasularisation, early onset of graft dysfunction based on Quick's value, as well as the use of a refined scoring-system for initial graft function based on a multi-parameter (AST, ALT, Quick and bilirubin) score according to Heise and co-workers [19].

Moreover, pathophysiological, molecular analyses will be performed as to investigate the impact of hypothermic reconditioning on hepatocellular autophagy [13,20] and early pro-inflammatory surface activation (ICAM-1, TLR expression) after reperfusion.

### Sample size calculation

The sample size calculation is performed for the primary endpoint (maximum absolute AST value during the first three days after transplantation) which is analyzed by an exact two-sided Mann-Whitney-U-test. Under the null hypothesis we expect no differences of the primary endpoint distributions for the two groups which translates into a relative effect *p *= 0.5, i.e. it is similarly likely to observe larger/smaller maximum AST values under one or the other group condition. Under the alternative hypotheses larger maximum AST values are on average expected for the standard treatment. For a significance level α = 0.05 (two-sided) and 52 patients per group would be necessary for a power of 0.8 to detect a relative effect of *p *= 0.66 (which is comparable to a mean difference of ~0.6 in units of standard deviations of a standard normal distribution if a parametric location shift (t-test) model would be appropriate). Considering a drop out rate of 10%, 58 patients per group (overall 116 patients) will be required.

### Randomization and treatment

The randomization will be a stratified 1:1 stratified block randomization with blocks of variable block length with patient-level stratification factor Model for End-Stage Liver Disease (MELD) score (three levels: < 20, 20-30 und > 30). The donor liver is randomized by our study design. Only patients who had given informed consent to participate in the study will be included in the randomization and the study.

Randomization will be technically realized by a web-interface as organized by the Center for Clinical Trials Essen (ZKSE) who will also train the staff in running the software. Moreover, the ZKSE will monitor and control the randomization.

Treatment is restricted to the isolated liver graft after arrival in the transplantation clinic. In the treatment group, donor livers arriving at the hospital will be subjected to 2 hours of venous systemic oxygen persufflation (OP) prior to implantation, while still being stored in ice cold preservation solution as described previously [7,16,21].

In brief, medical grade oxygen gas is passed through a wash bottle and filtered through a membrane with pore size of 5 μm inserted into the inflow line. The filtered and humidified gas is then introduced into the liver by connecting the tubing to a catheter, previously inserted into the suprahepatic caval vein. In order to avoid any kind of barotraumas to the vulnerable hepatovasculature, The applied driving pressure of the gas entering the liver is limited to 18 mmHg, e.g. by means of an interposed pressure relief valve.

**Figure 1:**
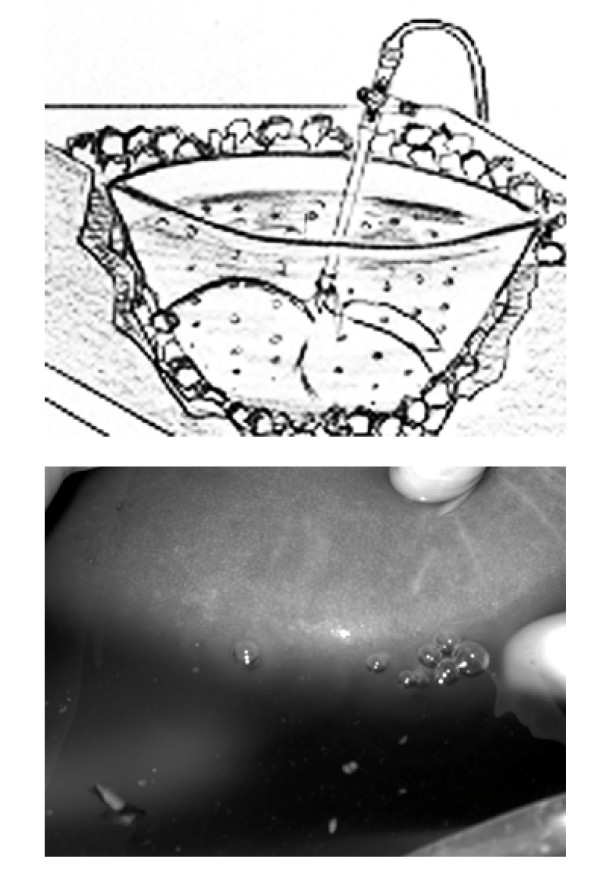
**Hypothermic reconditioning by gaseous O_2_-persufflation**. Above: Schematic representation of  the persufflated organ during cold storage on ice   Below: Detail of gas bubbles leaving a human liver through small pin-pricks set into post-sinusoidal venules.

The liver is continuously placed immerged in cold preservation solution in a beaker, kept cold by surrounding crush ice (cf. Figure 1).

The infrahepatic caval vein is temporarily closed by means of a small atraumatic bulldog clamp. After connecting the persufflation catheter to the gas tubing, postsinusoidal venules appear to be dilated due to the pressure applied to the hepatic venous system. Using a small 27 gauge syringe needle, small pinpricks are set into the dilated venules in all areas at the periphery of the liver lobes that allow the gas to leave the microvasculature.

After completion of the preparative procedures, sterile surgical gauze is soaked with the preservation solution and used to cover the liver during the time of persufflation.

Patients randomized to the control group receive a liver graft kept simply cold stored until implantation.

### Ethical considerations

The responsible physician will inform the patient about the background and present knowledge on the treatment protocol under study. It must be emphasized that the patient is allowed to refuse the treatment at any time prior to the operation. Before the patient is entered in the study the patient's written consent will be obtained. The principal investigator will ensure that this study will be carried out in agreement with the "Declaration of Helsinki [22] and local legal and regulatory requirements. The trial is monitored by the ZKS Essen according to Standard Operation Procedures (SOP) that are based on ICH-GCP guidelines.

An independent safety board monitors closely the proper conduct of the trial and all SAE reports to ensure the safety of the subjects during the course of the study.

The study has been approved by the institutional ethical review committee.

### Statistical analyses and data management

The primary endpoint is the maximum absolute AST value (U/l) on one of the first three post-operative days. As the AST distribution is highly skewed a non-parametric statistical procedure, the two-sided exact Mann-Whitney-U-test, is used for the confirmatory analysis. A SAS Macro will be used to estimate the relative effect *p *and to calculate 95% confidence intervals. Though missing data issues pertaining to the primary endpoint should be minimized given the continued monitoring during study conduct, the possibly remaining missing data of the primary endpoint will be replaced by the maximum global (across both groups) AST values mimicking a "worst-case scenario". The impact of other replacement or imputation strategies will be analyzed in sensitivity analyses.

The confirmatory analysis will be performed for the intention-to-treat population (full-analysis set) which includes all patients randomized for whom transplantation has been initiated.

In addition, to address the non-parametric Behrens-Fisher-problem (the sensitivity of the Mann-Whitney-U-test to any differences in distributions including differences in variances) and to integrate the stratum variable "MELD-Score", sensitivity analyses are preplanned using ANOVA statistics.

All secondary endpoints (see above) will be analyzed descriptively as described in the statistics analysis plan.

Data management and statistical analyses will be performed by the Center for Clinical Trials Essen (ZKSE).

### Adverse events

Any unexpected clinical adverse event or abnormal laboratory test value that is serious, including death, occurring during the course of the study must be reported by the study coordinator Prof. Minor (Bonn) within 7 days using the Serious Adverse Event form. The investigator at the site Essen and Prof. Minor have to assess, if this SAE was (likely to be) caused by the transplantation and/or oxygen persufflation. This SAE will be sent to the IEC by Prof. Minor. The Data Safety Monitoring Board (DSMB) of OPAL will receive a list of SAE and complication twice a year. Cases of death will reported without delay to the DSMB.

## Discussion

This clinical trial is based on a long standing series of basic and translational research including classical mechanistic in vitro studies as well as in vivo experiments, optimizing and validating the innovative therapeutic approach of gaseous oxygen application on a pre-clinical level [9,11,13,21,23].

After having demonstrated its feasibility in a multi-case observation [16] it is now intended to establish a clinical proof of concept on the level of evidence of a randomized controlled, blinded clinical trial. However, due to the fact, that the surgeon inevitably gets aware of the pinpricks during the implantation procedure, it is not possible to do the trial in a double blinded fashion.

In our opinion it would seems unethical to include sham pinpricks in the standard procedure, for this would then no longer be a standard procedure. Moreover, side effects, however improbable, related to the presence of pin-pricks would not be as easily be attributed to the treatment group. We do not think, however, that the lack of blinding the surgeon will have a notable impact on the primary endpoint, i.e. the peak level of serum transaminases after transplantation.

Oxygen persufflation is an attractive adjunct in organ preservation, seeming particularly suitable in the preservation of the liver, which, unlike other organs, readily utilizes genuine glycogen as energetic substrate. While the therapeutic intervention can be postponed to the immediate pre-implantation period easily performed at any transplant center, gas persufflation will allow for re-equilibration of cellular signal and energy homeostasis and precludes the need of expensive or cumbersome disposables.

If the present study will confirm the conceptual expectations, a useful method to regenerate cellular energy dependent pathways in the graft is available to suppress injurious cellular activation cascades and to improve postischemic recovery of marginally preserved liver grafts.

## Competing interests

The authors declare that they have no competing interests.

## Authors' contributions

TM designed the study and drafted the manuscript. AP, CP, and CO co-authored the writing of the manuscript and contributed to the study design. All other authors participated in the design of the study during several meetings and are local sub-investigators. All authors read and approved the final manuscript.
